# Kenyan maize lepidopteran pests and microclimate monitoring dataset from 2021 to 2024

**DOI:** 10.1016/j.dib.2025.112006

**Published:** 2025-08-26

**Authors:** François Rebaudo, Judith Legrand, Paul-André Calatayud

**Affiliations:** a UMR EGCE, IRD, CNRS, Université Paris-Saclay, Gif-sur-Yvette, France; b UMR GQE - Le Moulon, Université Paris-Saclay, INRAE, CNRS, AgroParisTech, Gif-sur-Yvette, France; c icipe – P.O. Box 30772-00100, Nairobi, Kenya

**Keywords:** Corn, Insect, Climate, Pheromone, Temperature, Trap

## Abstract

The dataset consists of a network of five pheromone traps for the Kenyan maize lepidopteran pests *Busseola fusca, Chilo partellus,* and *Spodoptera frugiperda*, together with associated microclimatic data. Traps were placed along an altitudinal gradient from Lake Victoria to the Indian Ocean coast. Microclimatic data were acquired using sensors placed as close as possible to the traps, with temperature, relative humidity and atmospheric pressure recorded for each site. Its potential for re-use is applied in agronomy and ecology for the study of interactions between pest species along a climatic gradient, and in the light of the recent invasion of the pest *Spodoptera frugiperda*.

Specifications Table

**Table:** 

Subject	Biology
Specific subject area	*Population dynamics and interactions between major maize pest in Eastern Africa, Kenya, along with microclimatic data.*
Type of data	Raw, Table, CSV files.
Data collection	Pest data were collected manually from pheromone traps in five locations in Kenya from Lake Victoria to the Indian Ocean coast. Microclimatic data were collected using either commercial sensors (Hobo MX2301A) or microclimatic stations (BME680 and SI1145 sensors from Adafruit; DS18B20 sensors from Maxim Integrated), connected to single-board computers (Raspberry Pi 3A+). Climatic dataset consists of temperature, relative humidity, and atmospheric pressure (with visible, IR and UV light in some trap location). Data were collected at a minimum frequency of 15 min between 2022 and 2024, and traps revised every day to every week. All date information was normalized to UTC.
Data source location	Country: Kenya, from Lake Victoria to the Indian Ocean coast : 3°25′33″S, 38°8′23″E; 3°26′48″S, 38°21′50″E; 0°26′12.45″S, 34°12′21.70″E; 1°16′12″S, 36°48′17″E; 4°20′38″S, 39°29′21″E
Data accessibility	Repository name: IRD dataverse; https://dataverse.ird.fr/ Data identification number: 10.23708/BDKV4U; 10.23708/IRNHFP; 10.23708/VYBAMQ; 10.23708/PFDYJQ; 10.23708/SM7CWG Direct URL to data: https://doi.org/10.23708/BDKV4U; https://doi.org/10.23708/IRNHFP; https://doi.org/10.23708/VYBAMQ; https://doi.org/10.23708/PFDYJQ; https://doi.org/10.23708/SM7CWG
Related research article	none

## 1 Value of the Data

• Trapping data provide valuable information on the population dynamics of the main maize pests in an area where this crop is of prime importance for food security.
• Combined with microclimatic data including temperature, relative humidity, and atmospheric pressure at a high frequency of 15 min over several years, these data are of relevance for ecology and agronomy to study the relationship between pest performance and environmental variables, but also to study interactions between species along an altitudinal gradient.
• This dataset, running from 2021 to 2024, is also an opportunity for a broad audience of researchers to study the effect of microclimate and potential climatic hazards on pest population dynamics in East Africa.

## 2 Background

Maize *Zea mays* L. is a key crop in Kenya of prime importance for food security, even more in the context of global change [1]. Maize production is expanding, putting greater pressure on land, with consequences for crop protection [2]. Historically, the key pests of maize have been lepidopteran stem-borers: the spotted stem borer *Chilo partellus* and the maize stalk borer *Busseola fusca* [3]. The year 2016 saw the arrival of the fall armyworm *Spodoptera frugiperda* from Americas. Since its invasion of Kenya, this species has been the main threat to maize cultivation [4], while at the same time upsetting the balance of agroecosystems [[5], [6], [7]]. Although experiments have been carried out to understand species interactions with regards to maize resource utilization and models have been built to predict the evolution of these populations in a context of global warming [8], documenting the population dynamics of these three pests in the field remains critical for a better understanding of their life cycles in relation to environmental variables. This monitoring also represents an essential source of data for validating existing models, or those that will be developed in the future, to ensure sustainable protection of maize crops in Kenya and more widely in East Africa.

## 3 Data Description

**Fig. 1:**
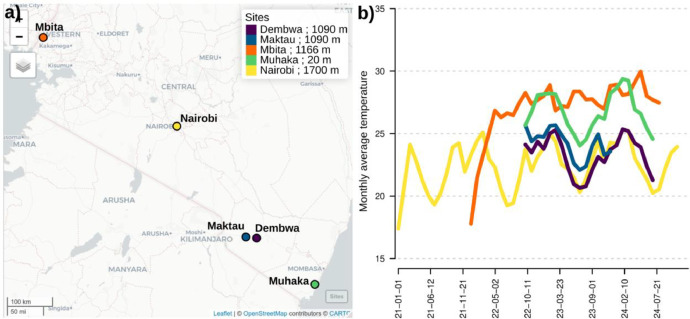
Location of the study sites and representation of the climatic dataset. a) Kenya’s map with the five sites from Victoria lake to the Inland (Nairobi, Maktau and Dembwa) and in Ocean coast (Muhaka). b) monthly average temperatures for all sites.

**Fig. 2:**
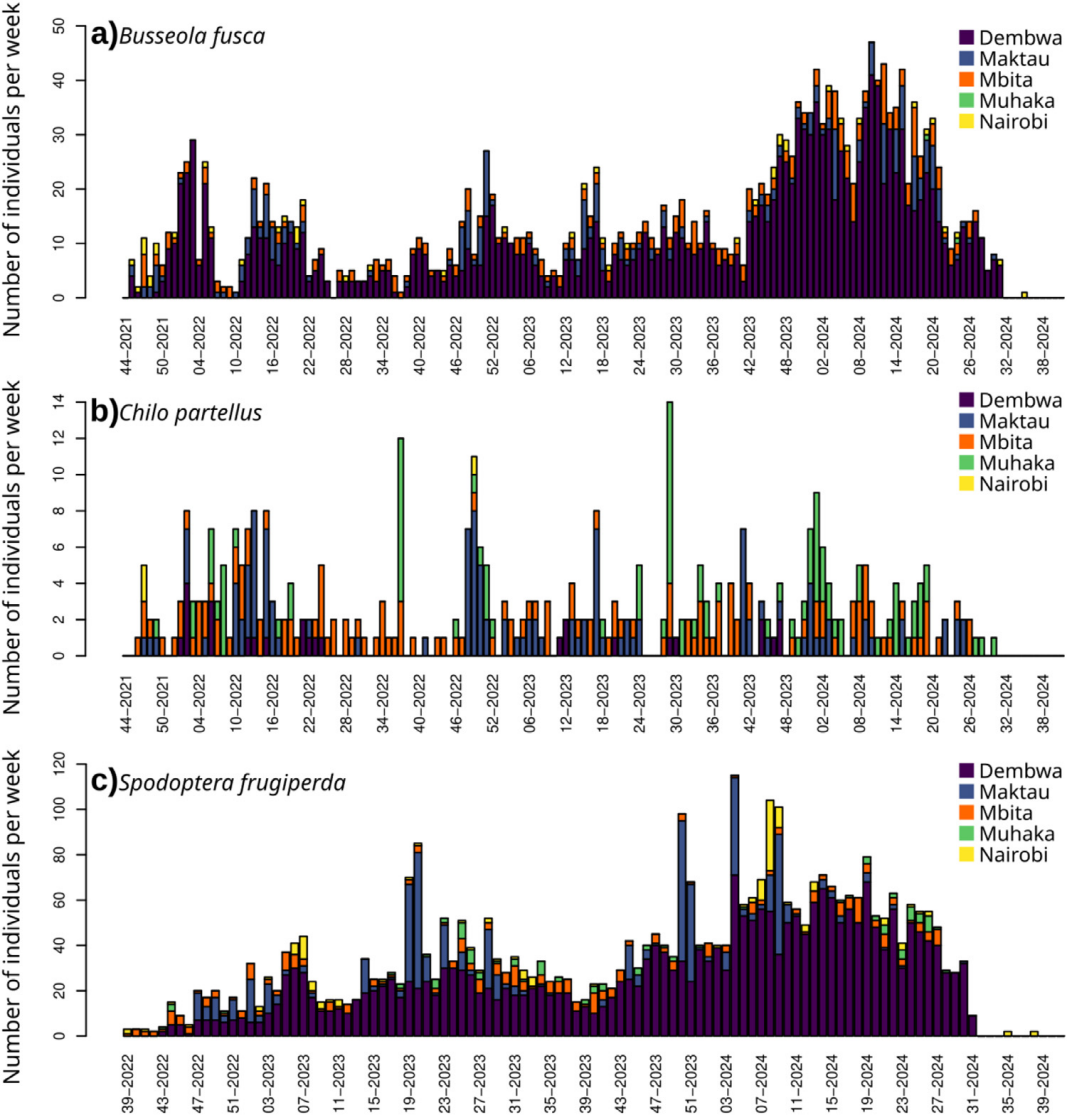
Number of individuals per site and per week for the three species a) *Busseola fusca*, b) *Chilo partellus*, c) *Spodoptera frugiperda.*

The files associated with this data-in-brief article includes microclimatic data and trap monitoring data for the five monitored sites, all in Comma Separated Value (CSV) file format. This dataset has given rise to several deposits in the dataverse so as to credit the various people who contributed to the data collection, which differs from one site to another [[9], [10], [11], [12], [13]]. All files start with “bdd_kenya_” followed by the site name, and the type of content (“bf” for *Busseola fusca* monitoring, “cp” for *Chilo partellus* monitoring, “sp” for *Spodoptera frugiperda* monitoring, and “climate” for the microclimatic dataset). All pest monitoring files have six columns for site, date, number of individuals caught since last survey date (the first date corresponds to the trap installation date), pheromone renewal date, and comments (Table 1). The climatic datasets differ from one site to another. All devices were positioned at crop canopy height, about 1 m above ground level. In Taita Hills, Dembwa and Taita Hills, Maktau (bdd_kenya_dembwa_taitaHills_climate.csv and bdd_kenya_maktau_climate.csv), temperature, relative humidity and dew point were recorded every 30 min. In Mbita Points, and Msambweni Kwale (bdd_kenya_mbitaPoints_climate.csv and bdd_kenya_muhaka_rpi_climate.csv), temperature, relative humidity ( %RH), atmospheric pressure, visible, ultraviolet and infrared light were recorded every 10 min. Additionnal sensors were used in Msambweni Kwale with temperature, relative humidity and dew point were recorded every 30 min (bdd_kenya_muhaka_field_climate.csv and bdd_kenya_muhaka_lab_climate.csv). In Nairobi (bdd_kenya_nairobi_climate.csv), temperature, relative humidity and atmospheric pressure were recorded every minute (Table 2, Fig. 1, and Fig. 2).

**Table 1:** Monitoring files and content for each species and site. #individuals corresponds to the total number of captures over the monitoring period. #obs corresponds to the total number of trap records over the study period.

File name	Site	Species	Start date	End date	#individuals	#obs.
bdd_kenya_dembwa_taitaHills_bf.csv	Taita Hills, Dembwa	B. fusca	2021–11–07	2024–07–31	1574	995
bdd_kenya_dembwa_taitaHills_cp.csv	Taita Hills, Dembwa	C. partellus	2021–11–07	2024–07–31	27	995
bdd_kenya_dembwa_taitaHills_sf.csv	Taita Hills, Dembwa	S. frugiperda	2022–10–22	2024–07–31	2536	646
bdd_kenya_maktau_bf.csv	Taita Hills, Maktau	B. fusca	2021–11–14	2024–07–28	254	140
bdd_kenya_maktau_cp.csv	Taita Hills, Maktau	C. partellus	2021–11–14	2024–07–28	120	140
bdd_kenya_maktau_sf.csv	Taita Hills, Maktau	S. frugiperda	2022–10–22	2024–07–28	585	91
bdd_kenya_mbitaPoints_bf.csv	Mbita Points	B. fusca	2021–11–16	2024–07–08	269	805
bdd_kenya_mbitaPoints_cp.csv	Mbita Points	C. partellus	2021–11–16	2024–07–08	134	805
bdd_kenya_mbitaPoints_sf.csv	Mbita Points	S. frugiperda	2022–09–29	2024–07–08	287	539
bdd_kenya_muhaka_bf.csv	Msambweni, Kwale	B. fusca	2021–11–04	2024–07–31	2	750
bdd_kenya_muhaka_cp.csv	Msambweni, Kwale	C. partellus	2021–11–04	2024–07–31	96	754
bdd_kenya_muhaka_sf.csv	Msambweni, Kwale	S. frugiperda	2022–10–18	2024–07–31	98	459
bdd_kenya_nairobi_bf.csv	Nairobi	B. fusca	2021–11–08	2024–10–09	48	1070
bdd_kenya_nairobi_cp.csv	Nairobi	C. partellus	2021–11–08	2024–10–09	3	1070
bdd_kenya_nairobi_sf.csv	Nairobi	S. frugiperda	2022–09–27	2024–10–09	128	747

**Table 2:** Climatic files and content for each site. RH % corresponds to relative humidity. #obs corresponds to the number of time points at which climatic data were recorder over the monitoring period.

File name	Site	Variables	Start date	End date	#obs.
bdd_kenya_dembwa_taitaHills_climate.csv	Taita Hills, Dembwa	temperature, RH %, dew point	2022–10–21	2024–07–31	31,177
bdd_kenya_maktau_climate.csv	Taita Hills, Maktau	temperature, RH %, dew point	2022–10–22	2023–12–17	20,208
bdd_kenya_mbitaPoints_climate.csv	Mbita Points	temperature, RH %, pressure, visible, UV and IR light	2022–01–21	2024–08–16	78,989
bdd_kenya_muhaka_field_climate.csv	Msambweni, Kwale	temperature, RH %, dew point	2022–10–19	2024–07–31	31,280
bdd_kenya_muhaka_lab_climate.csv	Msambweni, Kwale	temperature, RH %, dew point	2022–10–19	2024–03–19	24,822
bdd_kenya_muhaka_rpi_climate.csv	Msambweni, Kwale	temperature, RH %, pressure, visible, UV and IR light	2022–10–19	2024–05–29	69,390
bdd_kenya_nairobi_climate.csv	Nairobi	temperature, RH %, pressure	2021–01–13	2024–11–08	3420,066

## 4 Experimental Design, Materials and Methods

The pest monitoring data were acquired using classical funnel traps equipped with sexual pheromones from pherobank (The Netherlands; https://www.pherobank.com; product codes 50,197, 50,038, and 50,047 for *S. frugiperda, B. fusca*, and *C. partellus*, respectively), specific to each monitored species (one trap per species and per site, with a technical team trained to recognize the three species). Observations were made manually by technical staff in Kenya. Traps were located within maize parcels at the edge of the plot, approximately one meter above the ground for the whole duration of the monitoring (Taita Hills Maktau: 3°25′33″S, 38°8′23″E; Taita Hills Dembwa : 3°26′48″S, 38°21′50″E; Mbita Points: 0°26′12.45″S, 34°12′21.70″E; Nairobi: 1°16′12″S, 36°48′17″E; Msambweni Kwale Muhaka : 4°20′38″S, 39°29′21″E).

The climatic dataset for Taita Hills Dembwa, Taita Hills Maktau, and Msambweni Kwale (Muhaka) were acquired using HOBO Temperature/RH Data Loggers MX2301A (LI-COR) which were located at the trap location, with a frequency of one measurement every 30 min. In Mbita Points, and also Msambweni Kwale (Muhaka), temperature, relative humidity, atmospheric pressure, visible, ultraviolet, and infrared light were recorded every 10 min from sensor modules connected to a Raspberry Pi 3A+ single-board computer from the nearest building, and powered with electrical socket. Temperature data were collected with both a DS18B20 sensor (Maxim Integrated) and a BME680 sensor (Bosch Sensortech) mounted in a module by Adafruit Industries. Relative humidity and atmospheric pressure were collected with the same BME680 sensor. Visible, ultraviolet and infrared light were recorded thanks to an SI1145 sensor from Adafruit Industries. Data were stored on a microSD card and sent to a server using a RESTful API [14]. In Nairobi, the same experimental design was used with a Raspberry Pi 3B+ to collect temperature, relative humidity, and atmospheric pressure from a BME680 Adafruit Industries module. Data acquisition was programmed on the Raspberry Pi computers under Raspibian and then Raspberry Pi OS, using python programming language [14].

All the data were compiled at the end of the monitoring period and standardized into Comma Separated Value (CSV) files type.

## Limitations

**Fig. 3:**
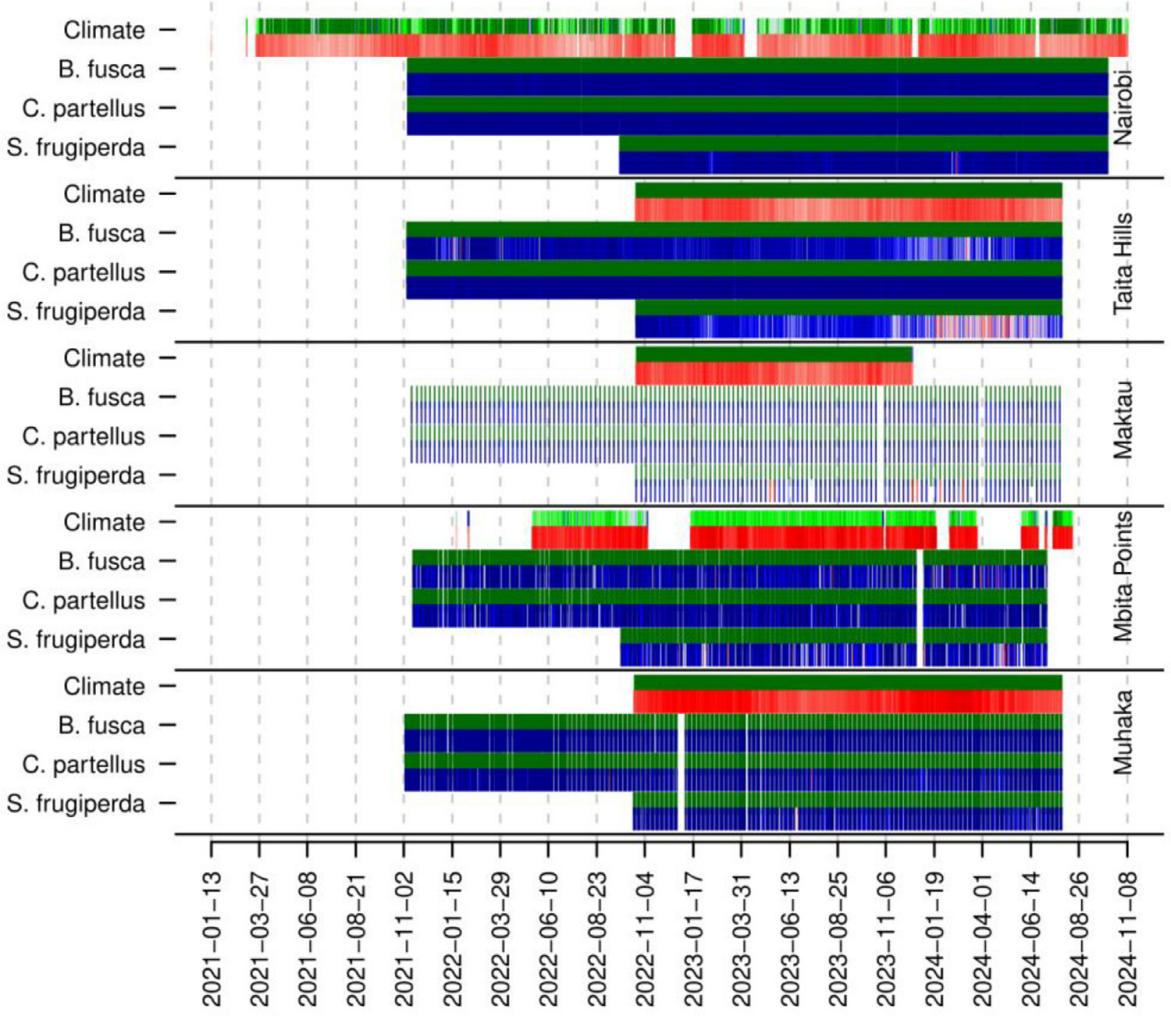
Schematic representation of the exhaustiveness of the data collected. The chart is divided into five rows for each monitoring site. Within each row, the temperature, *B. fusca, C. partellus*, and *S. frugiperda* monitoring is represented with two lines. The first line shows the frequency of observations (number of times data has been collected), with a gradient from white (no observations) to dark green (maximum observations). The second line provides an indication of the temperature measurement in the first row, and of the number of individuals captured in the following rows. A gradient from white to red is used for temperature (white for minimum temperatures and red for maximum temperatures), and a gradient from dark blue to white to red for insect captures (dark blue for absence of insects and red for maximum captures).

Insect collection data in the traps are subject to the availability of people to collect them, which can lead to certain irregularities in the surveys (e.g. particularly at Taita Hills Maktau), although in most cases the surveys were carried out on a daily basis (Fig. 3). For climatic data, readings based on single-board computers have sometimes been interrupted by power cuts (Fig. 3).

## Ethics Statement

The authors have read and follow the ethical requirements for publication in Data in Brief and confirm that the current work does not involve human subjects, animal experiments, or any data collected from social media platforms.

## CRediT authorship contribution statement

**François Rebaudo:** Conceptualization, Methodology, Resources, Writing – original draft, Visualization, Project administration, Funding acquisition, Writing – review & editing. **Judith Legrand:** Writing – review & editing. **Paul-André Calatayud:** Resources, Writing – review & editing.

## Data Availability

DataverseCorn pest and climate monitoring dataset from Msambweni, Kwale, Kenya, 2022–2024 (Original data). DataverseCorn pest and climate monitoring dataset from Nairobi, Kenya, 2022–2024 (Original data). DataverseCorn pest and climate monitoring dataset from Mbita points, Kenya, 2022–2024 (Original data). DataverseCorn pest and climate monitoring dataset from Taita Hill, Dembwa, Kenya, 2022–2024 (Original data). DataverseCorn pest and climate monitoring dataset from Taita Hill, Maktau, Kenya, 2022–2024 (Original data).

## References

[1] Mumo L., Yu J., Fang K. (2018). Assessing impacts of seasonal climate variability on maize yield in Kenya. Int. J. Plant Prod..

[2] De Groote H. (2002). Maize yield losses from stemborers in Kenya. Int. J. Trop. Insect Sci..

[3] Ong’amo G.O., Le Rü B.P., Dupas S., Moyal P., Calatayud P.-A., Silvain J.-F. (2006). Distribution, pest status and agro-climatic preferences of lepidopteran stem borers of maize in Kenya. Annales de La Société Entomologique de France (N.S.).

[4] De Groote H., Kimenju S.C., Munyua B., Palmas S., Kassie M., Bruce A. (2020). Spread and impact of fall armyworm (Spodoptera frugiperda J.E. Smith) in maize production areas of Kenya, agriculture. Ecosyst. Environ..

[5] Hailu G., Niassy S., Bässler T., Ochatum N., Studer C., Salifu D., Agbodzavu M.K., Khan Z.R., Midega C., Subramanian S. (2021). Could fall armyworm, spodoptera frugiperda (J. E. Smith) invasion in Africa contribute to the displacement of cereal stemborers in maize and sorghum cropping systems. Int. J. Trop. Insect. Sci..

[6] Ntiri E.S., Calatayud P.-A., Van Den Berg J., Le Ru B.P. (2019). Spatio-temporal interactions between maize lepidopteran stemborer communities and possible implications from the recent invasion of spodoptera frugiperda (Lepidoptera: noctuidae) in Sub-Saharan Africa. Environ. Entomol..

[7] Mutyambai D.M., Niassy S., Calatayud P.-A., Subramanian S. (2022). Agronomic factors influencing fall armyworm (Spodoptera frugiperda) infestation and damage and its Co-occurrence with stemborers in Maize Cropping systems in Kenya. Insects.

[8] Régnier B., Legrand J., Calatayud P.-A., Rebaudo F. (2023). Developmental differentiations of major maize stemborers due to global warming in temperate and tropical climates. Insects.

[9] F. Rebaudo, P.-A. Calatayud, M. Mwadime, Corn Pest and Climate Monitoring Dataset from Taita Hill, Maktau, Kenya, 2022–2024, (2025). 10.23708/BDKV4U.

[10] F. Rebaudo, P.-A. Calatayud, J.J. Mwamburi, Corn Pest and Climate Monitoring Dataset from Taita Hill, Dembwa, Kenya, 2022–2024, (2025). 10.23708/IRNHFP.

[11] F. Rebaudo, P.-A. Calatayud, A. Odiwuor Owuor, Corn pest and Climate Monitoring Dataset from Mbita points, Kenya, 2022–2024, (2025). 10.23708/VYBAMQ.

[12] F. Rebaudo, P.-A. Calatayud, J.O. Obonyo, E. Mwangangi, Corn Pest and Climate Monitoring Dataset from Nairobi, Kenya, 2022–2024, (2025). 10.23708/PFDYJQ.

[13] F. Rebaudo, P.-A. Calatayud, B. Elesani, H.H. Vilio, Corn Pest and Climate Monitoring Dataset from Msambweni, Kwale, Kenya, 2022–2024, (2025). 10.23708/SM7CWG.

[14] Rebaudo F., Soulard T., Condori B., Quispe-Tarqui R., Calatayud P., Chavez Vino S., Tonnang H.E.Z., Bessière L. (2023). A low-cost IoT network to monitor microclimate variables in ecosystems. Methods Ecol. Evol..

